# Characterization of MATE-Type Multidrug Efflux Pumps from *Klebsiella pneumoniae* MGH78578

**DOI:** 10.1371/journal.pone.0121619

**Published:** 2015-03-25

**Authors:** Wakano Ogawa, Yusuke Minato, Hayata Dodan, Motoyasu Onishi, Tomofusa Tsuchiya, Teruo Kuroda

**Affiliations:** 1 Department of Microbiology and Biochemistry, Daiichi University of Pharmacy, Fukuoka, Japan; 2 Department of Microbiology, Graduate School of Medicine, Dentistry and Pharmaceutical Sciences, Okayama University, Okayama, Japan; 3 Department of Microbiology, College of Pharmaceutical Sciences, Ritsumeikan University, Shiga, Japan; University of Cambridge, UNITED KINGDOM

## Abstract

We previously described the cloning of genes related to drug resistance from *Klebsiella pneumoniae* MGH78578. Of these, we identified a putative gene encoding a MATE-type multidrug efflux pump, and named it *ketM*. *Escherichia coli* KAM32 possessing *ketM* on a plasmid showed increased minimum inhibitory concentrations for norfloxacin, ciprofloxacin, cefotaxime, acriflavine, Hoechst 33342, and 4',6-diamidino-2-phenyl indole (DAPI). The active efflux of DAPI was observed in *E*. *coli* KAM32 possessing *ketM* on a plasmid. The expression of mRNA for *ketM* was observed in *K*. *pneumoniae* cells, and we subsequently disrupted *ketM* in *K*. *pneumoniae* ATCC10031. However, no significant changes were observed in drug resistance levels between the parental strain ATCC10031 and *ketM* disruptant, SKYM. Therefore, we concluded that KetM was a multidrug efflux pump, that did not significantly contribute to intrinsic resistance to antimicrobial chemicals in *K*. *pneumoniae*. MATE-type transporters are considered to be secondary transporters; therefore, we investigated the coupling cations of KetM. DAPI efflux by KetM was observed when lactate was added to produce a proton motive force, indicating that KetM effluxed substrates using a proton motive force. However, the weak efflux of DAPI by KetM was also noted when NaCl was added to the assay mixture without lactate. This result suggests that KetM may utilize proton and sodium motive forces.

## Introduction

Bacterial drug efflux pumps have generally been classified into five families: the resistance nodulation cell division (RND) family, major facilitator superfamily (MFS), small multidrug resistance (SMR) family, ATP-binding cassette (ABC) family, and multidrug and toxic compound extrusion (MATE) family. Of these, proteins of the MATE family have been detected in Bacteria, Archaea, and Eukarya kingdoms [1].

MATE-type pumps were phylogenetically classified into three clusters by Brown et al [1]. NorM from *Vibrio parahaemolyticus* [2, 3], NorM from *Vibrio cholerae* [4], and YdhE from *E*. *coli* [2, 5] have been categorized into cluster 1 [1]. The antiport of substrates coupled with Na^+^ has often been reported as one of the characteristics of MATE-type transporters in cluster 1 [6]. NorM from *V*. *parahaemolyticus*, which was the first MATE-type transporter to be discovered, exhibited ethidium efflux activity using the electrochemical potential of Na^+^ [3]. Drug efflux activity coupled with Na^+^ was subsequently reported in YdhE from *E*. *coli*, VmrA from *V*. *parahaemolyticus* [7], and VcmA and VcrM from *V*. *cholera* non-O1 [8, 9]. PdrM, which we recently reported, also displayed sodium-driven drug efflux ability and was the first multidrug efflux pump coupled with Na^+^ to be identified in Gram-positive bacteria [10]. The efflux activity of substrates coupled with protons has been reported in AbeM from *Acinetobacter baumannii* [11], EmmdR from *Enterobacter cloacae* [12], PmpM from *Pseudomonas aeruginosa* [13], and PfMATE from *Pyrococcus furiosus* [14], which belong to cluster 1 in the MATE family.

Eukaryotic proteins in the MATE family from animal, plants, fungi, or yeast have been categorized into cluster 2 [1]. A phylogenetic analysis revealed that TT12 from *Arabidopsis thaliana* [15] and MATE transporters from mammals [16–18] belonged to this cluster [19, 20]. Previous studies reported that several transporters in this cluster antiported their substrates with protons [18, 19, 21, 22].

Proteins similar to DinF in *E*. *coli* have been included in cluster 3. Activity that elevated drug resistance was detected in the DinF homologues of several bacteria (*e*. *g*. MepA from *S*. *aureus* [23], VmrA from *V*. *parahaemolyticus* [7], and DinF from *Ralstonia solanacearum* [24]). Brown et al also demonstrated that virulence to a tomato cultivar was decreased in a *dinF*-disrupted mutant from *R*. *solanacearum* [24]. However, DinF from *E*. *coli* and most DinF homologues that we have investigated did not significantly change resistance levels to antimicrobial agents (our unpublished data and reference 10) [10]. DinF from *E*. *coli* was identified several decades ago and is known to be induced in response to DNA damage and belongs to the LexA-dependent SOS regulons [25, 26]. A previous study showed that DinF protectively functioned against oxidative stress and bile salts [27]. However, the physiological role of DinF remains unknown, even in *E*. *coli*.

In the present study, we characterized three MATE-type transporters from *K*. *pneumoniae*. One was categorized into cluster 1 and the others were categorized into cluster 3. One of these transporters exhibited DAPI efflux activity that was accelerated by sodium and potassium.

## Materials and Methods

### Bacterial strains, plasmids, and media

The bacterial strains and plasmids used in this study are listed in Table 1. *K*. *pneumoniae* MGH78578 was kindly provided by Dr. Michael McClelland of the Sidney Kimmel Cancer Center in San Diego, CA, USA. *K*. *pneumoniae* ATCC10031 was purchased from the American Type Culture Collection. *E*. *coli* KAM32 (Δ*acrB*, Δ*ydhE*) [7] was a drug-hypersusceptible strain. *K*. *pneumoniae* and *E*. *coli* were grown aerobically in L medium (1% polypeptone, 0.5% yeast extract, and 0.5% NaCl, pH 7.0) at 37°C. Cells were grown at 37°C under aerobic conditions in L medium, except for the measurement of the minimum inhibitory concentration (MIC). A total of 100 μg/mL ampicillin or 20 μg/mL chloramphenicol was added to L medium when needed. The growth of cells was monitored turbidimetrically at 650 nm. L-agar plates contained 1.5% agar.

**Table 1:** Bacterial strains and plasmids used in this study.

Strains and Plasmids		Reference
*K*. *pneumoniae*		
MGH78578	multidrug-resistant strain, used for the genome project	
ATCC10031	ATCC collection, the parental strain of SKY2 and SKYM	33
SKY2	deletion mutant of *acrAB* from ATCC10031	29
SKYM	deletion mutant of *ketM* from ATCC10031	This study
*E*. *coli*		
TG1	parental strain	
KAM32	deletion mutant of *acrB* and *ydhE*, cloning host	7
Plasmids		
pSTV28	vector	Takara Bio, Inc., Japan
pBluescript II SK(-)	vector	
pDSH8	pBluescript II SK(-) derivative, possessing functionally cloned *ketM*	This study
pNTV3	pSTV28 derivative carrying functionally cloned *ketM*	This study
pDEM2	pSTV28 derivative carrying PCR-cloned *ketM* with an original start codon sequence (GTG)	This study
pDEM2a	pSTV28 derivative carrying PCR-cloned *ketM*, whose start codon was modified to ATG	This study
pDEM3	pSTV28 derivative carrying PCR-cloned *dinF*	This study
pDEM4	pSTV28 derivative carrying PCR-cloned *yeeO*	This study

### Gene cloning

We previously described gene cloning procedures [28]. Candidates belonging to groups 3, 4, and 14 were from our previous study [28] and included the *ketM* gene.

### PCR cloning of putative MATE efflux pump genes

The coding region of *ketM* (ACCESSION YP_001335662.1), *dinF* (ACCESSION YP_001338054.1), or *yeeO* (ACCESSION YP_001338054.1) was amplified by PCR to construct pDEM2, pDEM2a, pDEM3, and pDEM4, respectively. pDEM2a carried *ketM*, the start codon of which was modified into ATG although the original start codon for *ketM* was GTG.

The primers used in this study were listed in Table 2. The conditions for PCR were 1 min at 95°C, 30 sec at 53°C, 1 min 30 sec at 68°C and repeated for 35 cycles. KOD-Plus DNA polymerase (TOYOBO Co. LTD., Osaka, Japan) was used for DNA amplification. The primers, ketMgtg-F and ketM-R were used to amplify the *ketM* coding region, dinF-F and dinF-R were used to amplify *dinF*, yeeO-F and yeeO-R were used to amplify *yeeO*, and ketM-F and ketM-R were used to amplify start-codon-changed *ketM* (Table 2). After being digested with *Sac* I and subjected to gel purification, each DNA fragment was ligated to pSTV28, which had been pre-digested by the same enzyme. After cloning, the absence of PCR-introduced errors in ORFs was confirmed by DNA sequencing.

**Table 2:** Primers used in this study.

Primers	Sequences	purpose
ketM 1	TAAGATGCACGCAGAAACGG	*ketM* gene disruption
ketM 2	GAAGCAGCTCCAGCCTACACAGCACGACCATGATCAGCAC	*ketM* gene disruption
ketM 3	CTAAGGAGGATATTCATATGGCTGACCGATATGATTGTGC	*ketM* gene disruption
ketM 4	TTTCGAACAATGGACCTGGG	*ketM* gene disruption
ketM FRT Fw	GTGCTGATCATGGTCGTGCTGTGTAGGCTGGAGCTGCTTC	*ketM* gene disruption
ketM FRT Re	GCACAATCATATCGGTCAGCCATATGAATATCCTCCTTAG	*ketM* gene disruption
ketM RT-F2	TTGGCAGAGAAGGCGGTTGG	RT-PCR
ketMRT-R3	CATGACCATCCCGGGCTTG	RT-PCR
dinF RT-F	TGGCGTCTTGCCCTCCCTATG	RT-PCR
dinF RT-R	CATGAAGAGGAAACTGGTGGCC	RT-PCR
yeeO RT-F	GCTGTACTGGCGCGAAATCAG	RT-PCR
yeeO RT-R	TTTTCCCAGCCAGCTGACAAGG	RT-PCR
uncB-F	GTCTGCTGTTCCTTGCCATG	RT-PCR
uncB-R	AGGTCCATCAGGTTCATCAG	RT-PCR
ketM-F	ATAAGAGCTCTTCGAAGGTGTTCACATG	*ketM* gene cloning
ketMgtg-F	ATAAGAGCTCTTCGAAGGTGTTCACGTG	*ketM* gene cloning
ketM-R	AAAAGCCCGCCGGAGGAGCTCGCTTAGACAAG	*ketM* gene cloning
dinF-F	GCGACGTGAGCTCTCAATCGAAGGTTCAAACATG	*dinF* gene cloning
dinF-R	CGGCAATGAGCTCAGATAACTAACTAAA	*dinF* gene cloning
yeeO-F	ATATCCAGAGCTCAAGAAGGTTTTGAAAT	*yeeO* gene cloning
yeeO-R	GAATTATTCTTCACTGAGCTCAAAAAATG	*yeeO* gene cloning

### MIC determination

The MICs of various antimicrobial agents were determined as described previously [29]. The same experiment was repeated at least five times and the most reproducible values were shown in Tables 3–6.

**Table 3:** Minimum inhibitory concentrations of various chemicals in *E*. *coli* cells transformed with functionally cloned *ketM*.

MIC (μg/mL)				
Antimicrobial Agents	Host cell: *E. coli* KAM32			
	pBluescript II(SK-)	pDSH8	pSTV28	pNTV3
Kanamycin	1	1	1	1
Streptomycin	2	2	2	2
Erythromycin	2	2	2	2
Tetracycline	0.25	0.25	0.25	0.25
Ciprofloxacin	<0.004	0.008	0.004	0.008
Nalidixic acid	1	1	1	1
Norfloxacin	0.016	0.125	0.016	0.063
Ofloxacin	0.016	0.016	0.016	0.016
Ampicillin	>2048	>2048	1	1
Aztreonam	0.125	0.125	0.06	0.06
Carbenicillin	>128	>128	4	4
Cefotaxime	0.016	0.03	0.016	0.008
Imipenem	0.125	0.125	0.125	0.125
Acriflavine	2	4	2	4
Chloramphenicol	0.5	0.5	64	64
DAPI	0.125	8	0.125	8
CTAB	16	16	16	16
Ethidium Br	2	2	2	2
Hoechst 33342	0.5	1	0.5	1
Rhodamine 6G	8	8	8	8
SDS	128	128	128	128

DAPI, 4',6-diamidino-2-phenyl indole; CTAB, hexadecyltrimethyl ammonium bromide; SDS, sodium dodecyl sulfate.

### Energy starvation and loading fluorescent substance

*E*. *coli* KAM32 harboring the recombinant plasmid was grown in L medium containing 100 μg/mL ampicillin at 37°C until OD_650_≈0.7. Cells were harvested and washed twice with modified Tanaka buffer (34 mM KH_2_PO_4_, 64 mM K_2_HPO_4_, 20 mM (NH_4_)_2_SO_4_, 0.3 mM MgSO_4_, 1 μM FeSO_4_, 1 μM ZnCl_2_, 0.01 mM CaCl_2_, pH 7.0) added at a final concentration of 2 mM MgSO_4_ [30]. Cells were resuspended in this buffer containing 4',6-diamidino-2-phenyl indole (DAPI, final concentration: 5 μM). Cells were incubated at 37°C for 2.5 hr in the presence of 5 mM 2,4-dinitrophenol (DNP) in order to load DAPI.

### DAPI efflux assay

The efflux of DAPI was performed as previously described [10]. Energy-starved and DAPI-loaded cells were washed three times with 0.1 M 3-morpholinopropanesulfonic acid (MOPS)-tetramethylammonium hydroxide (TMAH) (pH 7.0) containing 2 mM MgSO_4_ and 5 μM DAPI, and were then resuspended in the same buffer to make the cell suspension OD_650_≈0.4. Fluorometric measurements were performed at 37°C with a Hitachi F-2000 fluorescence spectrometer. Excitation and emission wavelengths were 332 nm and 462 nm, respectively. After being preincubated at 37°C for 4 min, lactate-TMAH (pH 7.0) (final concentration 20 mM) and salt were added to the assay mixture.

### RNA preparation and reverse transcriptase-polymerase chain reaction (RT-PCR) analysis

*K*. *pneumoniae* cells were harvested during the exponential phase of growth. Total cellular RNA was isolated from these cells using the Qiagen RNeasy Mini Kit (Qiagen Inc., USA). The cell lysate was treated with a QIAshredder column before loading onto the RNeasy column. An RNase-Free DNase (Qiagen Inc., USA) treatment was performed according to the manufacturer's protocol. Extracted total RNA was applied to RT-PCR with the QIAGEN One-Step RT-PCR Kit (Qiagen Inc., USA). The primers used for RT-PCR are listed in Table 2. PCR without the reverse-transcriptase reaction was performed to confirm the absence of detectable DNA contamination in the extracted RNA solution. RT-PCR was repeated five times with independently prepared template RNA and a set of reproducible result was shown in the result.

### Construction of *K*. *pneumoniae* SKYM

We previously described the method to disrupt a gene on the genome in *K*. *pneumoniae* [29, 31]. DNA fragments were amplified with the following primers: ketM 1, ketM 2, ketM 3, ketM 4, ketM FRT Fw, and ketM FRT Re (Table 2). pKD4 was used as a template for amplification with the primers ketM FRT Fw and ketM FRT Re. Chromosomal DNA from *K*. *pneumoniae* ATCC10031 was used as a template for amplification with the other sets of primers. A PCR reaction was then performed with the three kinds of PCR fragments to produce a fusion fragment.

### Computer-based analysis

Alignment and phylogenetic analyses of MATE-type proteins were performed with Clustal W (version 2.1 in DDBJ). Information on the proteins used for phylogenetic analyses were listed in S1 Table.

## Results

### Gene cloning

We previously reported the cloning of genes that elevated the resistance levels of host cells against antimicrobial compounds, and these genes were classified into seventeen groups based on the vector plasmids used for gene cloning, digestion pattern of restriction enzymes, and conferred drug resistance [28]. The partially determined DNA sequence of the insert of the hybrid plasmids categorized as groups 3, 4, and 14 were compared with the genome database for *K*. *pneumoniae* MGH78578 (http://www.ncbi.nlm.nih.gov/genome/815?project_id=57619), and we found that all cloned DNA fragments in groups 3, 4, and 14 possessed a common gene (ACCESSION YP_001335662.1, KPN_02001) encoding a predicted efflux pump classified into MATE family. We chose pDSH8 and pNTV3 for subsequent experiments because these plasmids possessed the shortest insert of the hybrid plasmids in groups 3, 4, and 14. The vector of pDSH8 was pBluescript II SK(-) while that of pNTV3 was pSTV28. The insert lengths of pDSH8 and pNTV3 were 2.5 kbp and 2.8 kbp, respectively, and these inserts did not include intact ORFs, except for the predicted MATE-type efflux pump gene. We designated the possible MATE-type efflux pump gene as *ketM*.

*ketM* encoded a protein similar to NorM from *V*. *parahaemolyticus* (ACCESSION AB010463.1). A comparison with the primary structure of KetM showed 85% identity and 96% similarity to YdhE (ACCESSION AAB47941.1) from *E*. *coli*, 56% identity and 86% similarity to NorM from *V*. *parahaemolyticus*, 54% identity and 86% similarity to VcmA (ACCESSION Q9KRU4.2) from *V*. *cholerae*, and 44% identity and 81% similarity to HmrM (ACCESSION P45272.1) from *Haemophilus influenzae* using the software, Genetyx Version 7.0.8. These proteins, which were homologous to KetM, were typical multidrug efflux pumps in the MATE family, and KetM was also thought to be a multidrug efflux pump in this family of cluster 1. Therefore, KetM was presumed to be categorized into cluster 1 in the MATE family.

### Drug susceptibility testing

The MICs of various antibiotics were measured in *E*. *coli* KAM32 transformed with the plasmid carrying *ketM* (Table 3). The MIC of norfloxacin in *E*. *coli* KAM32/pDSH8 was eight-fold higher than that of KAM32/pBluescript II SK(-). The MIC of norfloxacin in *E*. *coli* KAM32/ pNTV3 was four-fold higher than that of KAM32/pSTV28. In both strains carrying *ketM*, the MIC of DAPI was 64-fold higher than that of the control cells and the MICs of Hoechst 33342 and acriflavine increased two-fold. Therefore, *ketM* increased the resistance of the host cells to several antimicrobial chemicals with different structures. We speculated that these differences in MICs to several chemicals between KAM32/pDSH8 and KAM32/pNTV3 may be attributed to differences in the copy numbers of the plasmids in a host cell.

### DAPI Efflux Assay

KetM was thought to be a drug efflux pump in the MATE family. Several pumps belonging to cluster 1 in the MATE family were previously reported to be driven by the electrochemical potential of sodium [3, 7–9]. Meanwhile, AbeM from *A*. *baumannii*, which is also a drug efflux pump belonging to cluster 1 in the MATE family, appeared to be driven by the electrochemical potential of protons [11]. Therefore, we measured the efflux activity of DAPI in the presence or absence of sodium. The ionic radius of Li^+^ is similar to that of Na^+^ and can be regarded as an analogue of Na^+^. K^+^ is a congener of Na^+^. K^+^ also plays an important role in the formation of ΔΨ in the proton motive force; therefore, we investigated the effects of LiCl and KCl on DAPI efflux in addition to NaCl. Rb^+^ has been a utilized in a crystal structure analysis for MATE-type transporters [32]. However, we assumed that Na^+^ was unsuitable for a crystal structure analysis and used Rb^+^ instead. Therefore, we did not investigate the effects of RbCl on KetM.

**Fig 1:**
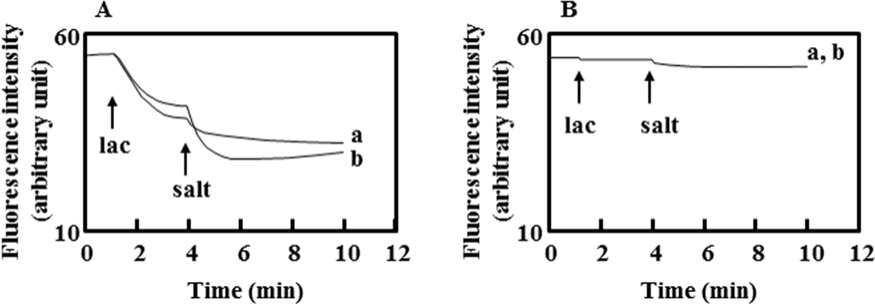
DAPI efflux activity was enhanced by NaCl and KCl. Lactate-TMAH (pH7.0) was added at a final concentration of 20 mM at the arrow point of lac. Panel A shows the results obtained with *E*. *coli* KAM32/pDSH8 and panel B shows those with *E*. *coli* KAM32/pBluescript II SK (-) as a control. A total of 20 mM NaCl (a) or 20 mM KCl (b) was added at the arrow point of the salt. Experiments were repeated four times and a representative of typical data was shown here.

**Fig 2:**
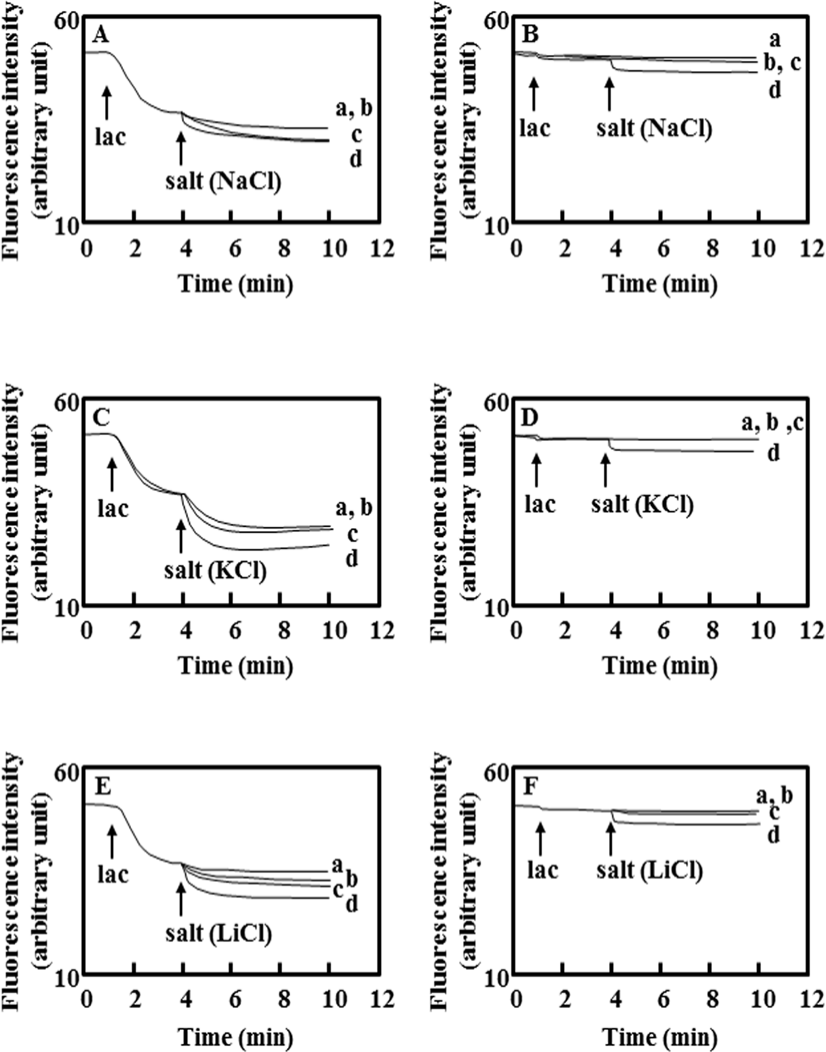
Salt concentration-dependent accelerations in DAPI efflux. Lactate-TMAH (pH7.0) was added at a final concentration of 20 mM at the arrow point of lac. Panels A, C, and E show the results obtained with *E*. *coli* KAM32/pDSH8 and panels B, D, and F were with *E*. *coli* KAM32/pBluescript II SK (-) as a control. NaCl was added in panels A and B, KCl was added in panels C and D, and LiCl was added in panels E and F, respectively, at the arrow point of the salt. The final concentrations of the salt were 1 mM (a), 5 mM (b), 10 mM (c), and 50 mM (d). The experiment for NaCl and KCl was repeated three times and while that for LiCl was repeated twice. A representative of typical data was shown here.

Energy-starved KAM32/pDSH8 showed stronger efflux activity than that of the control cells when the energy source was added (Fig. 1). This result indicated that DAPI was effluxed in an energy-dependent manner and the electrochemical potential of protons played a role in this efflux activity. The addition of NaCl stimulated the efflux of DAPI by KetM (Fig. 1). This was also observed with the addition of KCl or LiCl (Figs 1 and 2). The addition of KCl in particular strongly activated the efflux of DAPI.

We then investigated the dependency of the efflux of DAPI on salt concentrations (Fig. 2). DAPI efflux in *E*. *coli* KAM32/pDSH8 was accelerated by increases in NaCl concentrations, and reached a plateau at 50 mM NaCl. The similar result was obtained with KCl or LiCl.

These results indicated that KetM was an energy-dependent DAPI efflux pump. A proton motive force has been shown to drive the efflux activity of DAPI by KetM and KetM may efflux not only DAPI, but also other chemicals whose MICs were increased in *ketM*-carrying cells.

It was difficult to detect H^+^/norfloxacin antiport activity with our assay system, which used reverted membrane vesicles. An eight-fold increase in the MIC of norfloxacin was too weak to detect antiport activity. The results of MICs identified DAPI the best substrate to characterize the coupling cation of KetM. However, the fluorescence of DAPI itself disturbed the detection of fluorescence by the indicator for H^+^ movement.

**Fig 3:**
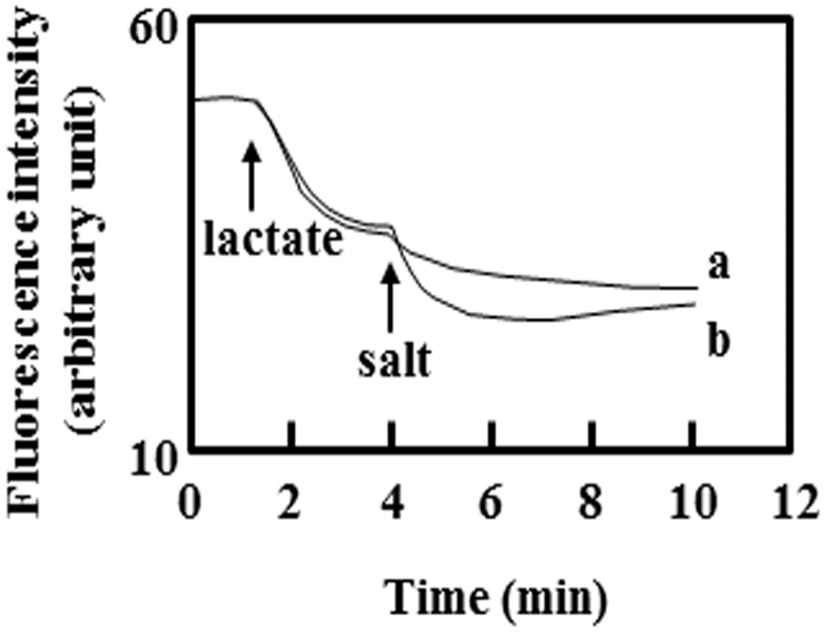
DAPI efflux was accelerated by cations. Lactate-TMAH (pH7.0) was added at a final concentration of 20 mM at the arrow point of lactate. A total of 20 mM Na_2_SO_4_ was added at the arrow point of the salt in curve a while 20 mM K_2_SO_4_ was added in curve b. The cells used for the assay were *E*. *coli* KAM32/pDSH8. The experiment was repeated twice and a representative of typical data was shown here.

Chloride did not appear to be important because a substitution with the sulfate ion instead of chloride did not change a DAPI efflux activity by KetM (Fig. 3).

### Measurement of MIC in the presence of NaCl or KCl

The efflux activity of DAPI by *ketM* was accelerated in the presence of Na^+^ or K^+^ (Fig. 1). We then investigated whether the MICs of norfloxacin, ciprofloxacin, DAPI, and kanamycin were increased in *E*. *coli* KAM32 carrying *ketM* in the presence or absence of 50 mM NaCl or 50 mM KCl. No significant changes were observed in the MICs of norfloxacin and ciprofloxacin in *E*. *coli* strains with the addition of the salts (S2 Table). The MICs of DAPI and kanamycin slightly increased (two-fold) with the addition of these salts in *E*. *coli* KAM32 carrying *ketM* (S2 Table). However, this slight increase in the MIC was also observed in *E*. *coli* KAM32 as the control (S2 Table), which suggested that the increase in the MIC in the presence of NaCl or KCl was not caused by KetM. Therefore, the acceleration of DAPI efflux activity by Na^+^ or K^+^ may not have been sufficiently effective to elevate the MICs of antibiotics.

### Contribution of KetM to intrinsic drug resistance

**Fig 4:**
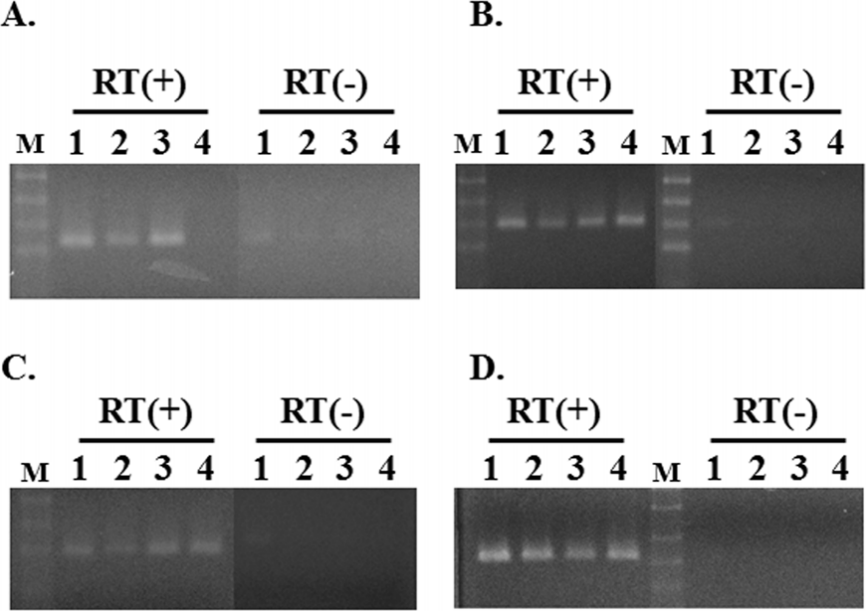
mRNA expression of deduced MATE-type transporter genes in *K*. *pneumoniae*. Gene expression was investigated by RT-PCR. Panel A: *ketM*, Panel B: *dinF*, Panel C: *yeeO*, and Panel D: *uncB*. The amplification of *uncB* was used as a standard control. Amplification was performed without a reverse-transcriptase reaction for samples in the RT (-) lanes. The experiment was repeated more than five times and the most reproducible result was shown. Lane 1: MGH78578, Lane 2: ATCC10031, Lane 3: NCTC9632, Lane 4: SKYM

The expression of mRNA was investigated in the three *K*. *pneumoniae* strains. *K*. *pneumoniae* MGH78578 is a highly drug-resistant strain that exhibits resistance to multiple antibiotics and antimicrobial chemicals. *K*. *pneumoniae* ATCC10031 possesses a nonsense mutation in *acrB* and its drug resistant levels were found to be markedly weaker than those of *K*. *pneumoniae* MGH78578 [33]. The measurement of MICs revealed that *K*. *pneumoniae* NCTC9632 had the intermediate drug-resistant levels of *K*. *pneumoniae* MGH78578 and ATCC10031 (Table 4). The expression of *ketM* mRNA was detected in all three strains (Fig. 4A); however, the expression levels of KetM did not parallel drug-resistant levels (Table 4).

**Table 4:** Minimum inhibitory concentrations of various chemicals in *Klebsiella* strains.

MIC (μg/mL)				
Antimicrobial Agents	MGH78578	ATCC10031	NCTC9632	SKYM
Kanamycin	2048	2	2	1
Ciprofloxacin	2	<0.008	0.03	0.008
Norfloxacin	8	0.06	0.125	0.06
Ampicillin	>1024	64	64	64
Aztreonam	>16	0.125	0.03	0.06
Cefotaxime	16	0.008	0.004	0.008
Acriflavine	512	8	64	8
DAPI	>128	>128	>128	>128
Ethidium Br	1024	32	256	32
Hoechst 33342	32	4	16	4

DAPI, 4',6-diamidino-2-phenyl indole

We disrupted *ketM* on the genome of *K*. *pneumoniae* ATCC10031 because the expression of its mRNA was detected. We designated this *ketM*-disrupted strain *K*. *pneumoniae* SKYM and tested its drug-resistant levels (Table 4). However, no significant changes were observed in any of the MICs tested between *K*. *pneumoniae* SKYM and the parental strain, ATCC10031. Therefore, the contribution to intrinsic drug resistance by KetM may be negligible in *K*. *pneumoniae*, at least under the conditions we used to measure MICs.

### Putative MATE efflux pumps in *K*. *pneumonaie* MGH78578

We identified three genes—*dinF* (KPN_04432), *yeeO* (KPN_02447), and *ketM*—that met the criteria of the MATE family by searching the *K*. *pneumoniae* genome database with comparisons to KetM and DinF from *E*. *coli*. We cloned these genes including *ketM* by PCR downstream of the *lac* promoter, and measured the MICs of several antibiotics in *E*. *coli* KAM32 and *K*. *pneumoniae* SKYM transformed with the cloned gene (Tables 5 and 6). The start codon of *ketM* was originally GTG while those of the other two genes were ATG, and we constructed two kinds of plasmids carrying *ketM* with GTG or ATG as the start codon.

**Table 5:** Minimum inhibitory concentrations of various chemicals in *E*. *coli* cells transformed with PCR cloned MATE transporters.

MIC (μg/mL)					
Antimicrobial Agents	Host cell: *E. coli* KAM32				
	pSTV28 (control)	pDEM2 (KetM)	pDEM2a (KetMATG)	pDEM3 (DinF)	pDEM4 (YeeO)
Kanamycin	0.5~1	1	0.5~1	0.5~1	0.5~1
Ciprofloxacin	0.004	0.004	0.004	0.004	0.004
Norfloxacin	0.03	0.03	0.03~0.06	0.03	0.03
Aztreonam	0.06~0.1	0.06	0.125	0.06	0.125
Cefotaxime	0.016	0.016	0.016	0.016	0.016
Acriflavine	2	2	4	2	2
DAPI	0.25	1	2	0.25	0.25
Ethidium Br	4	4	4	4	4
Hoechst 33342	0.5	0.5	0.5	0.5	0.5

DAPI, 4',6-diamidino-2-phenyl indole

**Table 6:** Minimum inhibitory concentrations of various chemicals in *K*. *pneumoniae* cells carrying putative MATE-type transporter genes on a plasmid.

MIC (μg/mL)					
Antimicrobial Agents	Host cell: *K. pneumoniae* SKYM				
	pSTV28 (control)	pDEM2 (KetM)	pDEM2a (KetMATG)	pDEM3 (DinF)	pDEM4 (YeeO)
Kanamycin	1	2	2	2	1
Ciprofloxacin	0.008	0.016	0.008	0.016	0.008
Norfloxacin	0.03~0.06	0.125	0.06	0.125	0.06
Ampicillin	64	64	64	64	64
Aztreonam	0.125	0.06	0.06	0.06	0.06
Cefotaxime	0.008	0.008	0.008	0.008	0.008
Acriflavine	8	8	8	8	8
DAPI	>128	>128	>128	>128	>128
Ethidium Br	32	32	32	32	32
Hoechst 33342	2~4	4	2	4	4

DAPI, 4',6-diamidino-2-phenyl indole

*E*. *coli* carrying *ketM* cloned by PCR also elevated resistance against DAPI even if the start codon was GTG or ATG (Table 5). pDEM2a carrying *ketM* with ATG as the start codon exhibited slightly stronger resistance to several chemicals than pDEM2. Functionally cloned *ketM* increased MICs more than PCR-cloned *ketM*. This result did not change when MICs were measured in the presence of IPTG.

The MICs of ciprofloxacin and norfloxacin were slightly increased by pNTV3 and pDEM2a in *K*. *pneumoniae* SKYM (Table 6). DAPI was considered to be a good substrate for KetM in *E*. *coli* KAM32, but the increase in the MIC for DAPI was not observed in *K*. *pneumoniae* SKYM. The MIC of DAPI may have originally been too high in *K*. *pneumoniae* SKYM to detect any MIC changes. Any notable change in this MIC was not observed when *E*. *coli* KAM32 and *K*. *pneumoniae* SKYM were transformed with pDEM3 or pDEM4. However, mRNA expression for *yeeO* and *dinF* were detected in *K*. *pneumoniae* under laboratory growth conditions (Fig. 4). This was similar to that observed for spr1756 and spr1877, which we previously described as MATE-type proteins in *S*. *pneumoniae* R6 [10]. The mRNA expression of these genes was detected in *Streptococcus pneumoniae* R6, whereas their artificial expression in *Bacillus subtilis* as a host cell did not cause MIC changes. Tocci et al reported that spr1756 was related to moxifloxacin resistance in *S*. *pneumoniae* R6, which was very weak [34]. We speculated that the main roles of *yeeO* and *dinF* also differed from the expulsion of antimicrobial chemicals.

## Discussion

In the present study, we characterized three MATE-type transporters from *K*. *pneumoniae*. One of the transporters, KetM elevated resistance to several antimicrobials and showed DAPI efflux activity. Therefore, it was considered to be a MATE-type multidrug efflux pump. However, cells transformed with *dinF* and *yeeO* deduced to encode the MATE-type transporter from *K*. *pneumoniae* MGH78578 did not exhibit increased drug resistance. Therefore, DinF and YeeO may have transported chemical compounds that were not investigated in the present study.

Resistance to both norfloxacin and DAPI was increased in *E*. *coli* KAM32 transformed with *ketM*. NorM from *V*. *parahaemolyticus* was very similar to KetM. However, more substrate specificity and higher increases in drug-resistant levels were observed when *norM* from *V*. *parahaemolyticus* was introduced into *E*. *coli* KAM32 [2]. YdhE from *E*. *coli* was also markedly similar to KetM but did not cause marked increases in drug resistance that were observed with *norM* from *V*. *parahaemolyticus* [2, 35]. This slight increase in drug resistance by YdhE was not attributed to the problems associated with promoter recognition or translation efficiency because the host cell was the same *E*. *coli* as the cloned *ydhE*. *K*. *pneumoniae* and *E*. *coli*, belonging to *Enterobacteriaceae*, may not strongly depend on MATE-type transporters for self-defense against antimicrobials. This prospect appeared to be supported by *K*. *pneumoniae* SKYM and *E*. *coli* KAM32 not showing any changes to drug sensitivity [7].

A comparison of the primary structure of KetM identified that this protein belonged to cluster 1 of MATE-type multidrug efflux pumps. NorM from *V*. *parahaemolyticus* [2, 3], which was the first MATE-type transporter discovered, has been categorized into cluster 1 [1]. MATE-type multidrug efflux pumps are thought to utilize Na^+^ or H^+^ as a coupling cation and more MATE-type transporters coupling with Na^+^ have been reported than transporters coupling with H^+^ in cluster 1. The transport of DAPI by KetM was facilitated by the addition of NaCl. Previous studies showed that the activity of transporters coupling with Na^+^ were accelerated in the presence of NaCl [36, 37]; therefore, we expected to detect the movement of Na^+^ by KetM with the addition of norfloxacin. We, however, could not detect it (S1 Fig).

KetM was able to efflux DAPI without Na^+^ (Figs 1, 2, and 3), and Na^+^ did not appear to be indispensable for this efflux activity. This characteristic differed from that of NorM from *V*. *parahaemolyticus* [3].

**Fig 5:**
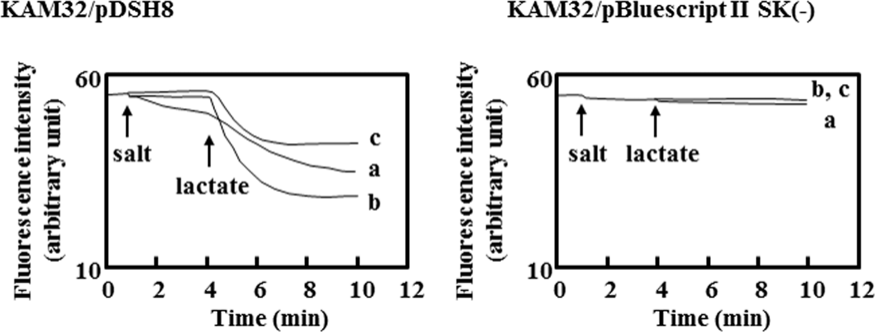
DAPI efflux activity when the salt was added prior to lactate. A total of 20 mM NaCl (a) or 20 mM KCl (b) was added prior to lactate-TMAH at the arrow point of the salt. The same volume of H_2_O was added as a control (c). Lactate-TMAH (pH7.0) was added at a final concentration of 20 mM at the arrow point of lactate. The experiment was repeated twice and a representative of typical data was shown here.

To complicate matters further, DAPI transport activity was enhanced by the addition of Na^+^, K^+^, or Li^+^ in the presence of an energy source (lactate) (Fig. 2). We assumed that the acceleration with Na^+^ and that with the other two kinds of cations should be considered separately because the addition of only NaCl without lactate facilitated the efflux of DAPI by KetM. On one hand, the addition of only KCl or LiCl without lactate could not transport DAPI (Fig. 5). These results suggested that a proton motive force may be indispensable for facilitating substrate transport for K^+^ and Li^+^, whereas Na^+^ itself could transport the substrate of KetM. NorM from *V*. *cholerae* is considered to be a MATE-type transporter that utilizes the electrochemical potential of Na^+^. However, van Veen et al recently reported that NorM from *V*. *cholerae* also utilized a proton motive force to transport ethidium [38]. The efflux of ethidium by NorM from *V*. *cholerae* was facilitated in the presence of Na^+^ and this transporter could only efflux ethidium with the addition of glucose. The phenomenon observed with KetM appeared to be similar to that with NorM from *V*. *cholerae*. Therefore, we suggested that the utilization of cations (H^+^ and Na^+^) by KetM may resemble that by NorM from *V*. *cholerae*.

**Fig 6:**
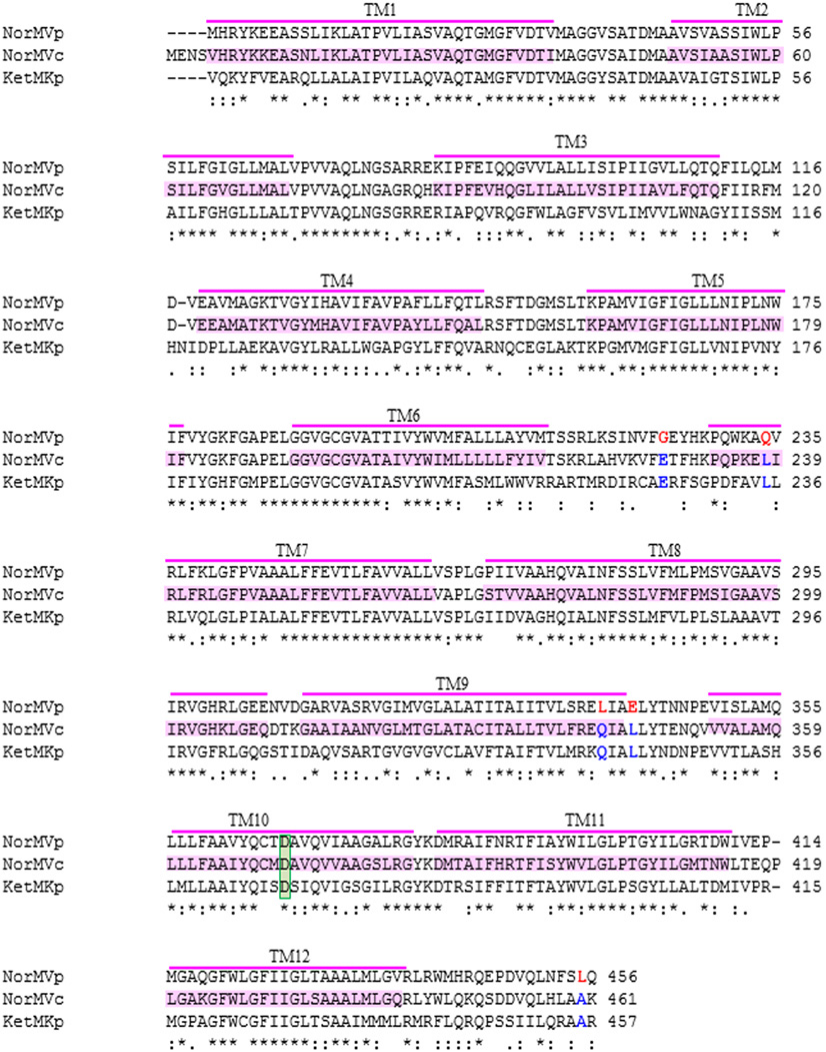
Alignment of NorM from *V*. *parahaemolyticus*, NorM from *V*. *cholerae*, and KetM from *K*. *pneumoniae*. NorM from *V*. *parahaemolyticus* (NorMVp, Accession: BAC59742.1), NorM from *V*. *cholerae* (NorMVc, Accession: EKY33397.1), and KetM from *K*. *pneumoniae* (KetMKp, Accession: ABR77432.1) were aligned (Clustal W version 2.1 in DDBJ). The transmembrane (TM) domains deduced from the 3D structure of NorM from *V*. *cholerae* are shown in pink. Amino acid residues that were common between NorM from *V*. *cholerae* (NorMVc) and KetM from *K*. *pneumoniae* (KetMKp) only, and deduced to be structurally different from NorM from *V*. *parahaemolyticus* (NorMVp) were marked in blue (NorMVc and KetMKp). As a reference, the corresponding amino acid residues in NorM from *V*. *parahaemolyticus* were marked in red. Asp367 in NorM from *V*. *parahaemolyticus*, Asp371 in NorM from *V*. *parahaemolyticus*, and Asp368 in KetM from *K*. *pneumoniae* were marked in green. Asterisks indicate amino acids that were common to all sequences at a particular position. Colons and dots indicate structurally similar amino acids as calculated by the Clustal W program.

We compared the primary structure of KetM from *K*. *pneumoniae* to those of NorM from *V*. *parahaemolyticus* and *V*. *cholerae*, and searched for common amino acid residues between NorM from *V*. *cholerae* and KetM from *K*. *pneumoniae* only. Nineteen amino acid residues were detected in this search, of which five amino acid residues were predicted to be structurally different from those of NorM from *V*. *parahaemolyticus*. Their positions were Glu225, Leu235, Gln339, Leu342, and Ala456 in KetM (Fig. 6). According to the 3D structure of NorM from *V*. *cholerae* (PDB: 3MKU), it was presumed that Glu225 was located in a loop between transmembrane domain (TM) 6 and TM7 in KetM. Leu235 was presumed to be in the end of TM7. Gln339 and Leu342 were presumed to be located in the end, close to the periplasm, in TM9. Ala456 was located in the C-terminal on the cytosol side. The loop between TM6 and TM7 was located on cytosol side and appeared to wrap around the outside of TM3 and TM4 (PDB: 3MKU), predicted to be the lateral TMs in NorM from *V*. *cholerae*.

KetM was unsuitable for investigating whether these amino acid residues were related to the utilization of a proton motive force for substrate transport for several reasons: DAPI, the best substrate for KetM, was unsuitable for detecting the movement of H^+^ accompanied by substrate transport because of the fluorescence of DAPI. The fluorescence of DAPI disturbed the detection of the fluorescence of the pH indicator. The low solubility of DAPI in H_2_O was also problematic for measuring Na^+^ movement by KetM in our assay system, and MIC increases by norfloxacin were too low to detect H^+^ or Na^+^ movement. Furthermore, there were no options for substrates. Therefore, NorM from *V*. *cholerae* or *V*. *parahaemolyticus* was considered appropriate for identifying the amino acid residues that were important for cation recognition.

Information on crystal structures appears conclusive, and previous findings on crystals containing the Rb^+^ of NorM from *V*. *cholerae* strongly supported NorM from *V*. *cholerae* being a MATE-type transporter that utilizes the electrochemical potential of Na^+^ [32, 39]. However, a recent study reported that NorM from *V*. *cholerae* also utilized a proton motive force to transport ethidium [38]. They showed that the movement of H^+^ consequent to the addition of ethidium was lost by the substitution of Asp371 to Asn in NorM from *V*. *cholerae*, and suggested that this amino acid residue may be important for H^+^ transport [38]. We confirmed that these amino acid residues were also preserved in AbeM from *A*. *baumannii* (ACCESSION BAD89844.2) and PmpM from *P*. *aeruginosa* (ACCESSION AAG04750.1). However, we also showed that the amino acid residue corresponding to Asp371 in NorM from *V*. *cholerae* was widely preserved in MATE-type transporters driven by Na^+^ (*e*. *g*. Asp367 in NorM from *V*. *parahaemolyticus*, Asp367 in VcmA from *V*. *cholerae*, Asp373 in HmrM from *H*. *influenzae*, Asp364 in VcrM from *V*. *cholerae*, Asp362 in VmrA from *V*. *parahaemolyticus*, and Asp370 in PdrM from *Streptococcus pneumoniae*). The amino acid residue corresponding to Asp371 in NorM from *V*. *cholerae* was also preserved in KetM from *K*. *pneumoniae* (Asp368).

Otsuka et al previously reported that Asp367 in NorM from *V*. *parahaemolyticus* was important for substrate transport based on the findings obtained from the introduction of an artificial mutation at this site [40]. Based on these findings, we proposed that the importance of this amino acid residue cannot be limited to only one aspect (*e*.*g*. only coupling with Na^+^ or H^+^ or only the interaction or recognition of substrates) and may have multiple functions in the transport of substrates by MATE-type transporters. Otherwise, amino acid residues may play different roles in various bacteria.

KetM from *K*. *pneumoniae* transported DAPI with the addition of only lactate. NorM from *V*. *cholerae* also expelled ethidium with the addition of only glucose. We also observed a similar ethidium efflux pattern in a deduced MATE-type transporter from a *Serratia* species (manuscript in preparation).

**Fig 7:**
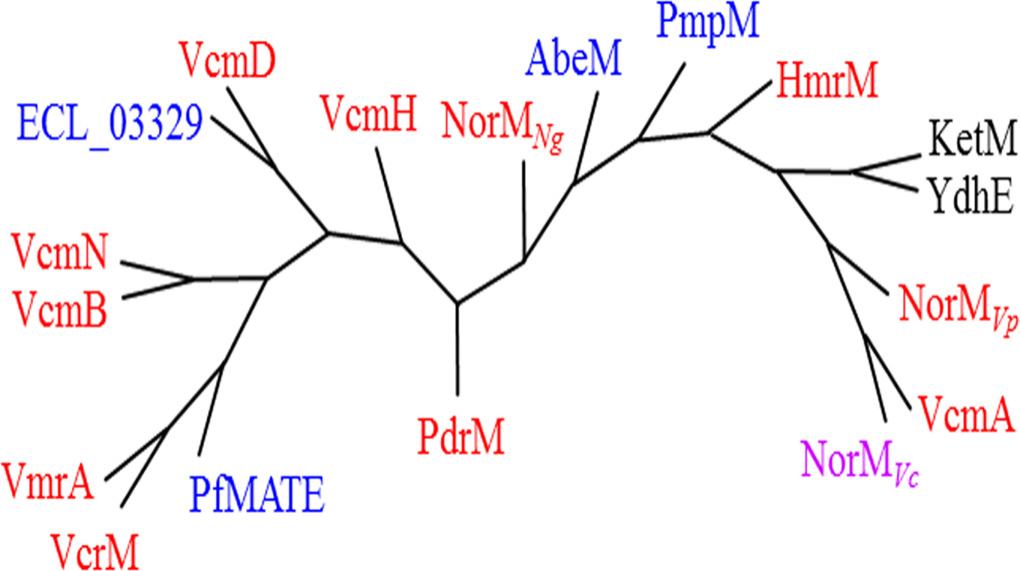
Phylogeny of KetM and MATE-type proteins in cluster 1. MATE-type proteins were analyzed with CLUSTAL W (the program used was ClustalW2.1). Na^+^-coupling MATE proteins were shown in red and H^+^-coupling MATE proteins were shown in blue. NorM_*Vc*_, which is considered to utilize both Na^+^ and H^+^, was shown in pink. KetM and YdhE, the coupling cations of which have not yet been identified, were shown in black. The criteria to determine cation utilization by each MATE-type transporter was shown in S1 Table. The accession number of each protein was follows; ABR77432.1 (KetM (*K*. *pneumoniae*)), AAB47941.1(YdhE (*E*. *coli*)), BAC59742.1 (NorM_*Vp*_ (*V*. *parahaemolyticus*)), Q9KRU4.2 (VcmA (*V*. *cholerae* non-O1)), EKY33397.1 (NorM_*Vc*_ (*V*. *cholerae* non-O1)), AB010463.1 (PdrM (*S*. *pneumoniae*)), WP_011011952.1 (PfMATE (*Pyrococcus furiosus*)), BAB70470.1 (VcrM (*V*. *cholerae* non-O1)), NP_798828.1 (VmrA (*V*. *parahaemolyticus*)), BAD98611.1(VcmB (*V*. *cholerae* non-O1)), BAD98614.1(VcmN (*V*. *cholerae* non-O1)), ADF62863.1 (ECL_03329 (*Enterobacter cloacae*)), BAD98612.1 (VcmD (*V*. *cholerae* non-O1)), BAD98613.1 (VcmH (*V*. *cholerae* non-O1)), AAW89139.1 (NorM_*Ng*_ (*Neisseria gonorrhoeae*), BAD89844.2 (AbeM (*A*. *baumannii*)), AAG04750.1 (PmpM (*P*. *aeruginosa*)), and P45272.1 (HmrM (*H*. *influenzae*)).

Collectively, these findings suggest the existence of three kinds of MATE-type transporters in bacteria; transporters that only utilize a sodium motive force, those that only utilize a proton motive force, and those that utilize sodium and proton motive forces. We conducted a phylogenetic analysis of MATE-type transporters in cluster 1, and found no correlation between cation utilization and phylogenetically-close proteins (Fig. 7). Therefore, an evolutionally apparent branching point to separate MATE-type transporters based on energy utilization did not appear to exist, and the assembly of partial structures (*e*.*g*. a cluster of amino-acid residues) may determine the cation selectivity of MATE-type transporters.

We could not identify the coupling cations of KetM directly in the present study. However, we showed that a proton motive force played an important role in the transport of substrates by KetM from *K*. *pneumoniae*.

## Supporting Information

S1 Fig Sodium movement with the addition of norfloxacin in *E*. *coli* cells transformed with *ketM*. We previously reported the details for this assay (PLoS One. 2013;8(3):e59525.). Briefly, cells were aerobically cultured in Tanaka medium containing 1% tryptone, 10mM melibiose, and 100 μg/ml ampicillin until the late exponential phase of growth at 30°C. These cells were then washed twice and suspended in 0.1 M 3-morpholinopropanesulfonic acid (MOPS)-tetramethylammonium hydroxide (TMAH) buffer. In the assay mixture, 0.1 M N-[Tris(hydroxymethyl)methyl]glycine (Tricine)-TMAH (pH8.0) containing) containing 33 μM NaCl was used. The final concentration of Methyl-β-D-thiogalactoside (TMG) was 5 mM while that of norfloxacin (NFLX) was 20 μM. These reagents were added at each arrow point. The detector marker drifted upwards when sodium influxed into the cell and moved downwards when sodium was antiported by a secondary added chemical (J Bacteriol. 2000;182(23):6694–6697). Sodium/NFLX antiport activity was not detected in *E*. *coli* cells transformed with pDSH8 even though the experiment to detect the sodium movement in this cell was performed six times. The sample cells were *E*. *coli* KAM32/pBluescript SK(-) (A) and *E*. *coli* KAM32/pDSH8 (B). The calibration control was drawn as the change from 60 nmol NaCl (C).
(TIF)

S1 Table MATE-type transporters whose cation utilization was investigated. (DOCX)

S2 Table Minimum inhibitory concentrations of various chemicals in the presence of salts in *E*. *coli* cells transformed with *ketM*. (DOCX)
