# Supplementary figures and images for: Characterization of MATE-Type Multidrug Efflux Pumps from Klebsiella pneumoniae MGH78578
Source: PLoS One. 2015 Mar 25;10(3):e0121619. doi: 10.1371/journal.pone.0121619 (PMC4373734; doi:10.1371/journal.pone.0121619)

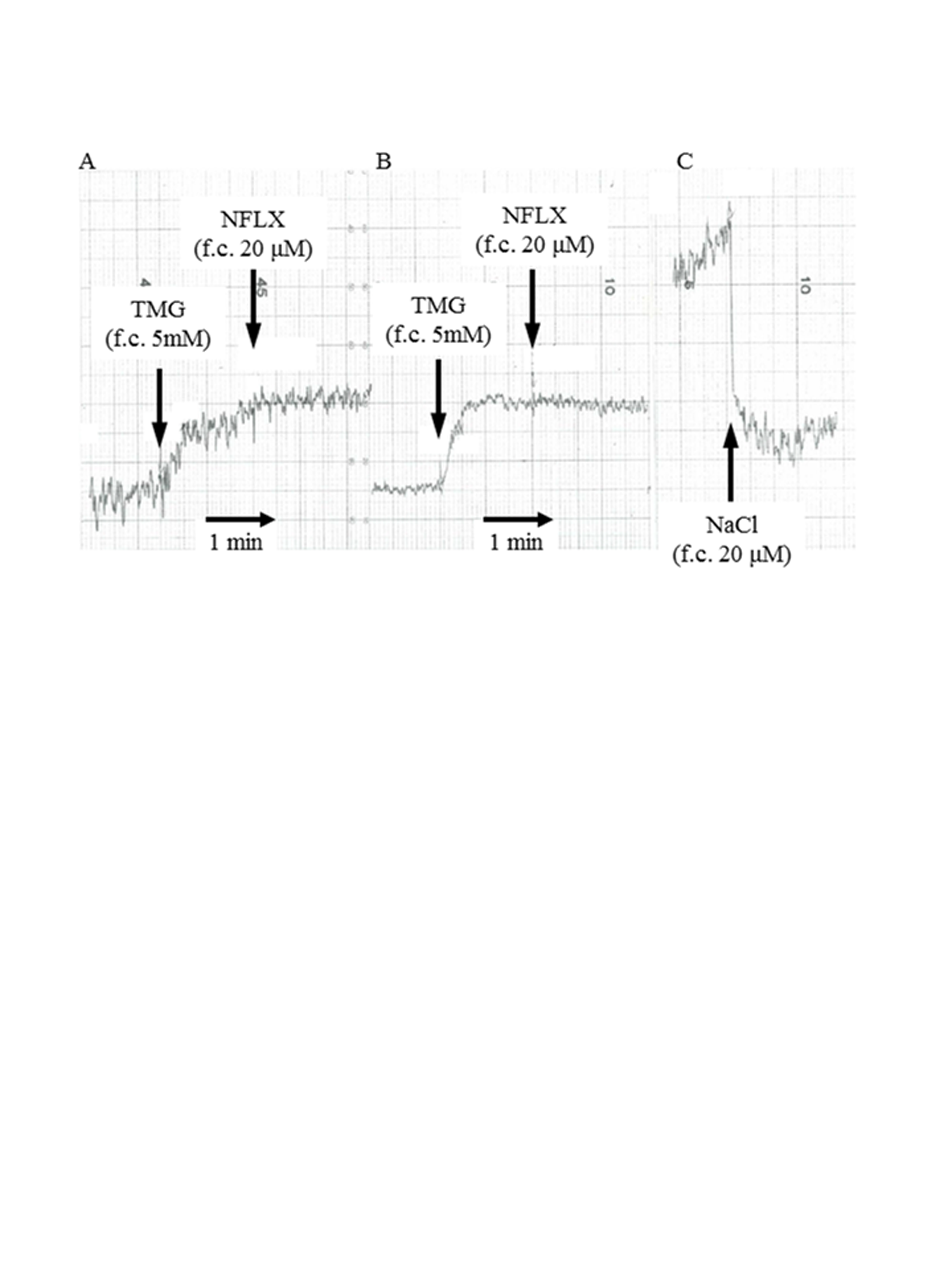

Supplement: S1 Fig — We previously reported the details for this assay (PLoS One. 2013;8(3):e59525.). Briefly, cells were aerobically cultured in Tanaka medium containing 1% tryptone, 10mM melibiose, and 100 μg/ml ampicillin until the late exponential phase of growth at 30°C. These cells were then washed twice and suspended in 0.1 M 3-morpholinopropanesulfonic acid (MOPS)-tetramethylammonium hydroxide (TMAH) buffer. In the assay mixture, 0.1 M N-[Tris(hydroxymethyl)methyl]glycine (Tricine)-TMAH (pH8.0) containing) containing 33 μM NaCl was used. The final concentration of Methyl-β-D-thiogalactoside (TMG) was 5 mM while that of norfloxacin (NFLX) was 20 μM. These reagents were added at each arrow point. The detector marker drifted upwards when sodium influxed into the cell and moved downwards when sodium was antiported by a secondary added chemical (J Bacteriol. 2000;182(23):6694–6697). Sodium/NFLX antiport activity was not detected in E. coli cells transformed with pDSH8 even though the experiment to detect the sodium movement in this cell was performed six times. The sample cells were E. coli KAM32/pBluescript SK(-) (A) and E. coli KAM32/pDSH8 (B). The calibration control was drawn as the change from 60 nmol NaCl (C). (TIF) [file pone.0121619.s001.tif]
