# Supplementary material for: Characterization of MATE-Type Multidrug Efflux Pumps from Klebsiella pneumoniae MGH78578
Source: PLoS One. 2015 Mar 25;10(3):e0121619. doi: 10.1371/journal.pone.0121619 (PMC4373734; doi:10.1371/journal.pone.0121619)
Supplement: S1 Table — (DOCX) [file pone.0121619.s002.docx]

| Supporting Information Table S1 MATE-type transporters whose cation utilization was investigated. | | | |  |
| --- | --- | --- | --- | --- |
| Protein | organism | coupling cation | reported evidence | reference |
| KetM | *K. pneumoniae* | ? | Not determined | this study |
| YdhE | *E. coli* | ? | Not determined | J Bacteriol. 2000, 182(23): 6694-7. Antimicrob Agents Chemother. 2008, 52(9): 3052–3060 |
| NorM | *V. parahaemolyticus* | Na^+^ | Not determined | J Bacteriol. 2000, 182(23): 6694-6697. |
| HmrM | *H. influenzae* | Na^+^ | Na^+^ efflux | Microbiol Immunol. 2003, 47(12): 937-43. |
| VcrM | *V. cholerae* non-O1 | Na^+^ | Na^+^ efflux | Microbiol Immunol. 2003, 47(6):419-427. |
| VmrA | *V. parahaemolyticus* | Na^+^ | Na^+^ efflux | J Bacteriol. 2002, 184(2):572-576. |
| VcmA | *V. cholerae* non-O1 | Na^+^ | Na^+^ efflux | FEMS Microbiol Lett. 2001, 203(2):235-239. |
| VcmN | *V. cholerae* non-O1 | Na^+^ | substrate efflux stimulated by NaCl | Microbiol Immunol. 2005, 49(11):949-957. |
| VcmH | *V. cholerae* non-O1 | Na^+^ | substrate efflux stimulated by NaCl |  |
| VcmB | *V. cholerae* non-O1 | Na^+^ | substrate efflux stimulated by NaCl | Microbiol Immunol. 2005, 49(11):949-957. |
| NorM | *Neisseria gonorrhoeae* | Na^+^ | substrate efflux stimulated by NaCl 3D structure with Cs^+^ | Antimicrob Agents Chemother. 2008, 52(9):3052-3060. Proc Natl Acad Sci U S A. 2013, 110(6):2099-104. |
| PdrM | *Streptococcus pneumoniae* | Na^+^ | Na^+^ efflux | PLoS One. 2013, 8(3):e59525. |
| VcmD | *V. cholerae* non-O1 | Na^+^ | substrate efflux stimulated by NaCl | Microbiol Immunol. 2005, 49(11):949-957. |
| NorM | *V. cholerae* | Na^+^, H^+^ | Na^+^: 3D structure with Rb^+^ H^+^: stimulation of ethidium efflux by an artificial pmf | Proteins. 2014, 82(2):240-249.  Biochemistry. 2013, 52(34):5790-5799 J Biol Chem. 2014, 289(21):14624-14632 |
| PfMATE | *Pyrococcus furiosus* | H^+^ | H^+^ movement with giant spheroplasts | Nature. 2013, 496(7444):247-251. |
| ECL_03329 | *Enterobacter cloacae* | H^+^ | H^+^ movement with inverted membrane vesicles | Arch Microbiol. 2011, 193(10):759-765. |
| AbeM | *Acinetobacter baumannii* | H^+^ | H^+^ movement with inverted membrane vesicles | Antimicrob Agents Chemother. 2005, 49(10):4362-4364. |
| PmpM | *P. aeruginosa* | H^+^ | H^+^ movement with inverted membrane vesicles | J. Bacteriol. 2004, 186 (1):262-265 |
