# Supplementary material for: Characterization of MATE-Type Multidrug Efflux Pumps from Klebsiella pneumoniae MGH78578
Source: PLoS One. 2015 Mar 25;10(3):e0121619. doi: 10.1371/journal.pone.0121619 (PMC4373734; doi:10.1371/journal.pone.0121619)
Supplement: S2 Table — (DOCX) [file pone.0121619.s003.docx]

**Supporting Information Table S2**

**Minimum inhibitory concentrations of various chemicals in the presence of salts in *E. coli* cells transformed with *ketM***

|  | KAM32/pSTV28 | | | KAM32/pNTV3 | | | KAM32/pBluescript SK(-) | | | KAM32/pDSH8 | | |
| --- | --- | --- | --- | --- | --- | --- | --- | --- | --- | --- | --- | --- |
|  | - | +50mM NaCl | +50mM KCl | - | +50mM NaCl | +50mM KCl | - | +50mM NaCl | +50mM KCl | - | +50mM NaCl | +50mM KCl |
| DAPI | 0.25 | 0.25-0.5 | 0.5 | 8 | 8 | 8-16 | 0.25 | 0.5 | 0.5 | 8 | 8-16 | 8-16 |
| Km | 1 | 2-4 | 2 | 1 | 2 | 4 | ND | ND | ND | ND | ND | ND |
| NFLX | 0.032 | 0.032-  0.064 | 0.064 | 0.064-  0.128 | 0.064 | 0.064 | 0.032-  0.064 | 0.032-  0.064 | 0.032-  0.064 | 0.128 | 0.128 | 0.128 |
| CFLX | 0.008 | 0.008 | 0.008 | 0.008 | 0.008-  0.016 | 0.008-  0.016 | 0.004-  0.008 | 0.004-  0.008 | 0.004-  0.008 | 0.008-  0.016 | 0.008-  0.016 | 0.016 |

DAPI, 4',6-diamidino-2-phenyl indole; Km, kanamycin; NFLX, norfloxacin; CFLX, ciprofloxacin; ND, not determined

This experiment was repeated four times and the most reproducible values were shown.
